# Prussian Blue Scavenger Ameliorates Hepatic Ischemia-Reperfusion Injury by Inhibiting Inflammation and Reducing Oxidative Stress

**DOI:** 10.3389/fimmu.2022.891351

**Published:** 2022-05-25

**Authors:** Yongxin Huang, Qinyuan Xu, Jiang Zhang, Yanze Yin, Yixiao Pan, Yuanyi Zheng, Xiaojun Cai, Qiang Xia, Kang He

**Affiliations:** ^1^ Department of Liver Surgery, Renji Hospital, School of Medicine, Shanghai Jiao Tong University, Shanghai, China; ^2^ Shanghai Engineering Research Center of Transplantation and Immunology, Shanghai, China; ^3^ Shanghai Institute of Transplantation, Shanghai, China; ^4^ Department of Ultrasound in Medicine, Shanghai Jiao Tong University Affiliated Sixth People’s Hospital, Shanghai, China

**Keywords:** prussian blue, liver ischemia-reperfusion injury, inflammation, reactive oxygen species, macrophage polarization

## Abstract

Oxidative stress and excessive inflammatory responses are the two critical mechanisms of hepatic ischemia-reperfusion injury (HIRI) encountered in many clinical settings, including following hepatectomy and liver transplantation. Effective anti-inflammatory and anti-oxidative pharmacological interventions are urgently needed to counter HIRI. The present study showed that a biocompatible Prussian blue (PB) scavenger with reactive oxygen species (ROS) scavenging and anti-inflammatory properties might be used a promising treatment for HIRI. Following intravenous administration, PB scavenger was mainly distributed in the liver, where it showed excellent ability to alleviate apoptosis, tissue injury and organ dysfunction after HIRI. PB scavenger was found to protect liver tissue by scavenging ROS, reducing neutrophil infiltration and promoting macrophage M2 polarization. In addition, PB scavenger significantly reduced oxidative stress in primary hepatocytes, restoring cell viability under oxidative stress condition. PB scavenger effectively reduced lipopolysaccharide-stimulated inflammation in RAW 264.7 cells. These findings indicate that PB scavenger may be a potential therapeutic agent for the treatment of HIRI, providing an alternative treatment for ROS-associated and inflammatory liver diseases.

## 1. Introduction

Ischemia-reperfusion injury (IRI) is a pathophysiological condition, in which organs and/or tissue experience hypoxia damage caused by the impairment of blood flow, followed by exacerbation of injury due to the restoration of blood circulation and oxygen delivery (1). Acute IRI can occur in various tissue and organs throughout the body, leading to tissue damage and dysfunction. The liver, being the largest solid organ in the human body, is likely to experience IRI in patients undergoing liver transplantation or hepatectomy, or in patients affected by hemorrhagic shock or liver injury (2). The process of hepatic IRI involves a series of events, with inflammation and excessive reactive oxygen species (ROS) being the most critical factors contributing to apoptosis, tissue injury and organ dysfunction (3).

At present, the strategies for treatment of HIRI are limited to ischemic preconditioning (IPC), ischemic post-conditioning (IPostC) and machine perfusion, with no pharmacological interventions available to protect the liver from IRI (4–7). Because the pathophysiology of hepatic IRI involves various targets and mechanisms, many types of drugs are currently being tested for their ability to suppress IRI-associated damage and restore liver function (8).

Nanomaterials have unique properties, making their biological behaviors significantly different from those of conventional molecules, showing anti-cancer, anti-infection and antioxidation properties (9–11). Nanoscale drugs can enter capillaries, preferentially accumulating in mononuclear phagocyte systems (e.g., liver, spleen) and being taken up by Kupffer cells. This increases drug concentrations in injured liver tissue, enhancing their bioavailability and therapeutic efficacy. In addition, nanomaterials are more stable than conventional molecules in the circulatory system, with a longer half-life, allowing administered doses to be decreased and reducing side effects while maintaining efficacy. Aiming at the therapeutic targets of ROS and inflammation, bioactive nanomaterials including nanoceria (12), carnosic acid nanoparticles (13), and bilirubin nanoparticles have shown good therapeutic efficacy in the treatment of HIRI. Despite their efficacy, bioactive nanomaterials have several drawbacks that hinder their clinical translation. For example, nanoceria acting as a nano-antioxidant reduced HIRI injury by scavenging ROS, alleviating inflammation, and inhibiting activation of monocyte and macrophage cells (12). However, the biological activities of nanoceria are sensitive to their size, ratio of Ce^3+^/Ce^4+^ and active crystal surface (14–16), which is still a great challenge under exploration. In addition, the biosafety and their ROS scavenging property of nanoceria need further exploration (17). It is of great significance to develop an alternative bioactive nanodrug for efficient treatment of HIRI.

Prussian blue (PB) is a bioactive nanomaterial that has shown good biosafety and been approved by the U.S. Food and Drug Administration to treat exposure to radioactive/non-radioactive cesium and/or thallium (18). PB has shown multienzyme-like activities, including peroxidase (POD), catalase (CAT), and superoxide dismutase (SOD) activities, and acts as a good ROS scavenger. The antioxidant properties of PB can be attributed to its rich variable valence states in the structure of PB (such as Fe^3+^/Fe^2+^, [Fe(CN)_6_]^3-^/[Fe(CN)_6_]^4-^) (19). Furthermore, PB has shown good preventive and therapeutic efficacy in various ROS-related diseases, including in the treatment of ischemic stroke (20, 21) and neurodegeneration (22), the prevention of vascular restenosis after endovascular interventions (23), in skin wound healing (24), and in the treatment of inflammatory bowel disease (25). A biocompatible PB scavenger with ROS-scavenging and anti-inflammatory properties may show potential in the treatment of HIRI.

The present study tested the ability of PB scavengers to protect livers from IRI based on their outstanding ROS scavenging and anti-inflammatory properties. PB scavengers were prophylactic administered to a mouse model of IRI. These scavengers mostly accumulated in the liver and effectively alleviated IRI-associated liver damage. These findings indicated that PB scavengers had excellent ability to manage oxidative stress, as well as having immunomodulatory activities.

## 2. Material and Methods

### 2.1 Materials

Analytical grade Potassium ferricyanide (K_3_[Fe(CN)_6_]), polyvinylpyrrolidone (PVP, K30), and hydrochloric acid (HCl, 36.0%) were purchased from Sinopharm Chemical Reagent Co., Ltd.

### 2.2 Synthesis of PB Scavengers

In a typical experiment, K_3_[Fe(CN)_6_] (396 mg) and PVP (5 g) were dissolved and mixed in 1 M HCl (40 mL) at room temperature. After being stirred until clear, the solution was transferred to an oven and heated at 80°C for 24 h. The final product was collected by centrifugation and washed three times with deionized (DI) water, followed by dispersion in saline or lyophilization and storage at 4°C.

### 2.3 Characterization of PB Scavengers and Instruments

Sample morphology was assessed using a Zeiss Gemini 300 scanning electron microscope (SEM, Oberkochen, Germany). Transmission electron microscopy (TEM) and high-resolution TEM (HRTEM) images were obtained using a JEOL-2100F TEM (Tokyo, Japan). Hydrodynamic diameters and Zeta potentials were determined by dynamic light scattering (DLS, Malvern Zetasizer Nano-Z, Malvern, UK). X-ray diffraction (XRD) patterns were obtained on an X-ray diffractometer (Bruker D8 Advance, Karlsruhe, Germany). The surface valence of Fe was evaluated by X-ray photoelectron spectroscopy (XPS, Thermo Scientific K-Alpha, New York, NY, USA). Ultraviolet-visible (UV-vis) spectra were recorded using a PerkinElmer Lambda 750 spectrophotometer (Waltham, MA USA). Fourier transform infrared (FTIR) spectra were characterized using a Fourier Transform Infrared spectrometer (Nicolet iS20, Thermo Fisher Scientific). Raman spectroscopy was performed using a Renishaw Raman system (Renishaw inVia, London, UK). PB concentrations were determined by inductively coupled plasma atomic emission spectroscopy (ICP-AES) using a PerkinElmer Avio 200 system.

### 2.4 Catalase (CAT)-Like Activity of PB Scavengers

To assess the CAT-like activity of PB scavengers, 1.2 M hydrogen peroxide (H_2_O_2_) and 2.4 ug/mL PB were added to buffers at pH 5.5, 6.8 and 7.4, and the dissolved oxygen concentrations were measured for 10 min using a portable dissolved oxygen meter (INESA JPBJ-609L, China).

### 2.5 Hydroxyl Radicals (•OH) Scavenging Capacity of PB Scavengers

The ability of PB scavengers to eliminate •OH was assessed by electron spin resonance (ESR, Bruker MS5000, Germany) spectroscopy. Briefly, •OH was generated by mixing FeSO_4_ (50 mg/ml) and H_2_O_2_ (20 mM) and captured by the spin trap agent 5,5-dimethyl-1-pyrroline N-oxide (DMPO). Various concentrations of PB, DMPO and newly generated •OH were incubated for 8 min, and their ESR spectra were recorded at an X-ray microwave power of 10 mW, a modulation frequency of 100 kHz, a scan range of 3300–3450 G, and a modulation amplitude of 1 G.

### 2.6 Peroxidase (POD)-Like Activity of PB Scavengers

The POD-like activity of PB scavengers was tested using the chromogenic substrate 3,3′,5,5′-tetramethylbenzidine (TMB) in the presence of H_2_O_2_. Briefly, H_2_O_2_, TMB and PB scavengers at various concentrations were added to phosphate buffer saline (PBS) solutions of various pH. The POD-like behavior of PB was evaluated by recording the absorbance of oxidized TMB at 650 nm over time using a UV-vis spectrophotometer.

### 2.7 Animals

Male wild-type (WT) C57BL/6JGpt mice aged 8–10 weeks were purchased from Shanghai Model Organisms. All mice were housed under specific pathogen-free conditions in a temperature-controlled room (22–24°C) with normal light–dark cycle, and allowed food and water ad libitum. All animal protocols met the Guide for the Care and Use of Laboratory Animals (National Institutes of Health [NIH], Bethesda, MD, USA) and were approved by the Animal Use Board of the School of Medicine of Shanghai Jiao Tong University.

### 2.8 Establishment of a Mouse Hepatic Ischemia/Reperfusion Injury Model 

Male WT C57BL/6JGpt mice aged 8–10 weeks were randomly divided into two groups, a sham treatment and a HIRI group, with each group subdivided into two groups and pretreated with PBS or 1 mg/kg PB scavenger 24 h before the procedure. To induce partial hepatic IRI, mice were first anesthetized by intraperitoneal injection of 1% pentobarbital. After a midline laparotomy, the arterial/portal vessels to the cephalad lobes were clamped with microvascular clamps for 90 min. The peritoneum was sealed and covered with warm saline-soaked sterile gauze to avoid dehydration. Clamps were removed after 90 min to initiate reperfusion. Mice were sacrificed at 6 hours after reperfusion, and liver and serum samples were collected for further analysis. Sham groups underwent the same surgical procedures but without obstruction of blood vessels.

### 2.9 Serum Analysis

Serum ALT and AST levels were measured by ALT/AST kit (ThermoFisher, Waltham, MA, USA) according to the manufacturer’s instructions. Serum concentrations of TNF-α and IL-10 were measured using mouse TNF-α and IL-10 ELISA kits.

### 2.10 H&E Staining and TdT-Mediated dUTP Nick End Labelling Assay

Formalin-fixed liver tissue samples were dehydrated in graded alcohol, embedded in paraffin, and sectioned at 5-μm thickness. After deparaffinization, the sections were stained with hematoxylin and eosin (H&E) as routine protocols. Apoptotic cell death in liver tissue was analyzed by TUNEL staining.

### 2.11 Lipid Peroxidation Assay

Frozen liver tissue was homogenized in RIPA buffer and centrifuged. The MDA concentration in the homogenate was determined using a commercially available kit (Nanjing Jiancheng Bioengineering Institute, Nanjing, China), which measured thiobarbituric acid (TBA) reactivity. Briefly, the homogenate was mixed with trichloroacetic acid, the mixture was centrifuged and TBA was added to the supernatant. Lipid peroxidation was determined by measuring the red color of the solution at 532 nm with a spectrophotometer. Other procedures were performed according to the manufacturer’s protocols.

### 2.12 Assay of ROS Levels *In Vivo*

Fresh liver tissue samples (50 mg) were homogenized in 1 mL buffer, followed by centrifugation of the homogenate at 4°C for 10 minutes. A 190-μL aliquot of each supernatant was incubated with 10 μL ROS probe (BestBio, China) in each well of a 96-well plate at 37°C in the dark for 30 minutes. ROS levels were analyzed using fluorescence microplate reader at an excitation wavelength of 488 nm and an emission wavelength of 530 nm.

### 2.13 Real-Time PCR

Total RNAs were isolated with Trizol reagent (Sigma), followed by synthesis of cDNA using a PrimeScript Reverse Transcription Reagent Kit (Takara, Otsu, Shiga, Japan). A Step One Plus Real-Time PCR System (Thermo Fisher Scientific) and Sybr Premix Ex Taq Kit (Takara) were used for qPCR, with the expression of each target gene normalized to that of GAPDH. Genes were amplified using primers for *Tnf-α* (5′-CCTGTAGCCCACGTCGTAG-3′ [forward] and 5′-GGGAGTAGACAAGGTACAACCC-3′ [reverse]); *Il-1β* (5′-CTCCATGAGCTTTGTACAAGG-3′ [forward] and 5′-TGCTGATGTACCAGTTGGGG-3′ [reverse]); *Arg-1* (5′-CTCCAAGCCAAAGTCCTTAGAG-3′ [forward] and 5′-GGAGCTGTCATTAGGGACATCA-3′ [reverse]); *IL-10* (5′-CTTACTGACTGGCATGAGGATCA-3′ [forward] and 5′- GCAGCTCTAGGAGCATGTGG-3′ [reverse]); and *GAPDH* (5′-GAAATCCCATCACCATCTTCCAGG-3′ [forward] and 5′-GAGCCCCAGCCTTCTCCATG-3′ [reverse]). The levels of expression (fold change) of *Tnf-α*, *Il-1β*, *Arg-1*, and IL-10 mRNAs relative to that of *GAPDH* mRNA in liver tissue samples and RAW 264.7 cells were determined by the Ct (ΔΔCt) method.

### 2.14 MPO Activity Determination

Liver MPO activity was determined using an MPO Detection Kit (Nanjing Jiancheng Bioengineering Institute, China). Briefly, liver tissue was homogenized in 1 ml phosphate buffer (50 mM, pH 6.0) containing 0.5% hexadecyltrimethylammonium hydroxide and centrifuged at 12,000 r/min at 4°C for 20 min. A 10-μL aliquot of each supernatant was transferred to PBS (pH 6.0) containing 0.17 mg/mL 3,3′-dimethoxybenzidine and 0.0005% H_2_O_2_. The MPO activity of the supernatant was determined by measuring the H_2_O_2_-dependent oxidation of 3,3′-dimethoxybenzidine and normalized by measuring the total protein content in samples using a BCA protein assay kit.

### 2.15 Immunofluorescence Staining

Liver sections were deparaffinized, rehydrated, and blocked with 5% bovine serum albumin (BSA) for 1 hour at room temperature. After washing, the tissue slices were incubated overnight at 4°C with primary rabbit antibodies against F4/80 (1:5000, Servicebio), CD206 (1:400, Servicebio), and iNOS (1:200, Servicebio). After washing with PBS, the slices were incubated at room temperature for 1 h with goat anti-rabbit secondary antibodies labeled with HRP (1:500, Servicebio) or Cy3 (1:300, Servicebio). Nuclei were stained with DAPI (Sigma-Aldrich). Fluorescence images were obtained with a DM6B microscope (Leica Microsystems, Milan, Italy) and analyzed by ImageJ.

### 2.16 Isolation, Culture and Treatment of Primary Hepatocytes

Primary hepatocytes were isolated from livers as previously described (26). The isolated cells were cultured on dishes (3 × 10^6^ cells/6-cm dish), 6-well plates (2 × 10^5^ cells/well) or 96-well plates (1 x 10^4^ cells/well) in high-glucose Dulbecco’s modified Eagle’s medium (DMEM), supplemented with 1% pen-strep and 10% FBS at 37°C in an atmosphere containing 5% CO_2_. Cell viability was tested after treating primary hepatocytes with various concentrations of PB scavengers. The primary hepatocytes were also treated with various concentrations of H_2_O_2_ in the presence or absence of 50 μg/mL PB scavengers. Cell viability was analyzed by MTT assays (Sigma-Aldrich, St. Louis, MO, USA).

### 2.17 Assay of Intracellular Ferrous Iron Level

Intracellular levels of ferrous iron were determined using iron assay kits (#ab83366, Abcam). Briefly, primary hepatocytes were collected, washed with cold PBS, and homogenized in iron assay buffer, followed by the addition of iron reducer to the collected supernatant. An iron probe was added, and the samples were mixed and incubated for 1 hour. The optical density of each solution at 593 nm was immediately measured on a colorimetric microplate reader.

### 2.18 Intracellular ROS Measurements

Intracellular ROS generation was assessed using the ROS sensitive fluorescent probe DCFH-DA (Sigma-Aldrich). Briefly, primary hepatocytes were incubated with H_2_O_2_ (100 μM) for 16 h with or without PB scavengers. The cells were washed and incubated with 10 μM DCFH-DA for 30 min, with intracellular fluorescence visualized using a fluorescence microscope (TE2000, Nikon, Tokyo, Japan). To analyze the cellular ROS levels in RAW 264.7 cells, the cells were incubated with 10 μM of DCFH-DA at 37°C for 45 min in the dark, followed by washing, resuspension in HBSS and immediate analysis by flow cytometry.

### 2.19 *In Vivo* Biosafety of PB Scavengers

Male WT C57BL/6JGpt mice aged 8–10 weeks were randomly divided into two group, which were intravenously administered PBS (control group) or 1 mg/kg PB scavengers (PB nanozyme group) respectively through the tail vein. After 24 h, the mice were anesthetized and blood was collected from the venous sinus into an anticoagulant tube containing ethylenediaminetetraacetic acid (EDTA). The main organs of each mice were harvested, fixed and stained with hematoxylin and eosin (H&E). Routine blood tests were performed using standard procedures.

### 2.20 Statistical Analysis

Data were expressed as mean ± SEM. Results in two groups were compared by two-tailed t tests, whereas results in multiple groups were compared by one-way ANOVA. All statistical analyses were performed using GraphPad Prism software (version 8.0), with P values <0.05 considered statistically significant.

## 3. Results and Discussion

### 3.1 Synthesis and Characterization of PB Scavengers

**Figure 1:**
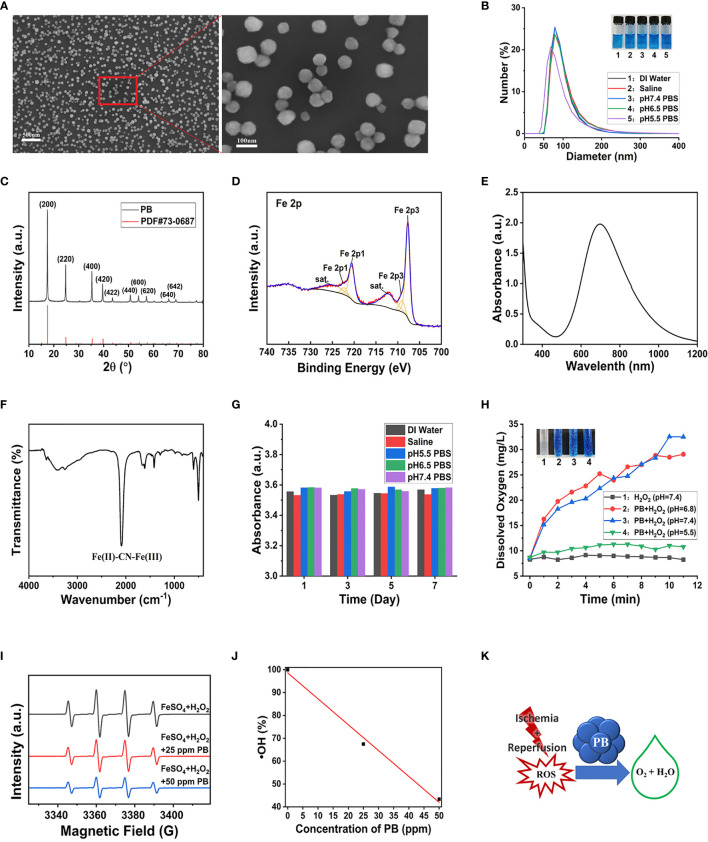
Characterization and multi-enzyme-like activity of PB. **(A)** SEM image of PB. **(B)** DLS determination of hydrodynamic diameter distribution. **(C)** X-ray diffraction patterns. **(D)** Fe 2p XPS spectrum. **(E)** Characteristic UV-vis peak of PB by UV–vis. **(F)** FT-IR spectra of PB. **(G)** Effect of incubation in different media for 7 days on the UV–vis absorbance of PB. **(H)** Rate of generation of dissolved oxygen (Inset: Digital photo of O_2_ bubbles generated from H_2_O_2_ 15 min after mixing the reagents). **(I)** ESR signal of DMPO/(FeSO_4_+ H_2_O_2_)-generated •OH. **(J)** ESR analysis of the •OH scavenging effect of different concentrations of PB (R^2 =^ 0.99275). **(K)** Schematic representation of the mechanism by which PB scavenges ROS.

PB scavengers were prepared as described (27, 28), by heating mixtures of K_3_[Fe(CN)_6_] and PVP dissolved in 1 M hydrochloric acid at 80°C for 24 h. In this synthesis process, PVP acted as a reducing agent under acidic conditions, as well as controlling and stabilizing size during lattice growth. The morphological features of PB scavengers were assessed by SEM and TEM, which showed that the constructed PB scavengers were approximately regular microspheres about 80.2 mm in diameter (**Figures 1A** and **S1**). Dynamic light scattering (DLS) showed that the mode hydrodynamic diameter of PB scavengers dispersed in various aqueous solutions was ~80 nm (**Figure 1B**). The crystallographic properties of PB were assessed by X-ray diffraction (XRD), which showed that the crystalline structure of PB perfectly matched the standard JCPDS card (73–0687) (**Figure 1C**) (29). X-ray photoelectron spectroscopy (XPS) substantiated the composition of PB The photoelectron spectroscopy of the Fe 2p orbit in PB. showed two split binding-energy peaks at 721.48 eV and 708.48 eV (**Figure 1D** and **S2**). PB scavengers showed a characteristic absorption peak at 700 nm due to the electron transition from Fe^II^ to Fe^III^ (**Figure 1E**). The chemical structure of PB was further evaluated by FTIR and Raman analysis. The FTIR spectrum showed a peak characteristic of a carbon-nitrogen triple bond (C≡N) at 2089 cm^−1^ (**Figure 1F**), whereas the remain spectrum showed a similar C≡N peak at 2155 cm^–1^ in the Raman spectrum (**Figure S3**). Zeta potentiometry showed that the zeta potential of PB was ~-13.7 V (**Figure S4**), indicating that the surface of PB scavenger was is negatively charged. The stability of PB *in vitro* was also investigated. Both UV-vis and DLS analyses showed that good dispersion of PB in various aqueous solutions, with no changes over 7 days of incubation (**Figures 1G**, **S5**). Taken together, these results confirmed that PB scavengers had been successfully synthesized.

### 3.2 Ability of PB Scavengers to Scavenge Multiple ROS

#### 3.2.1 CAT--Like Activity of PB Scavengers

CAT, which is synthesized in response to oxidative stimulation, is an important intracellular protective enzyme that catalyzes the decomposition of H_2_O_2_ to O_2_ and H_2_O (30). Thus, the rate of oxygen generation can reflect the catalase-like activity of PB scavengers. Measurements of the concentrations of dissolved oxygen produced over time by H_2_O_2_ and PB and recorded by the dissolved oxygen electrode showed that PB catalyzed the decomposition of H_2_O_2_ to generate abundant O_2_ bubbles at pH 6.8 and pH 7.5, but generated fewer bubbles under acidic conditions (pH 5.5) ( **Figure 1H**). By contrast, the control group without PB displayed no bubble generation. These findings indicated that PB scavengers exhibited CAT-like behavior through the decomposition of H_2_O_2_.

#### 3.2.2 •OH Scavenging Capacity of PB Scavengers

•OH was produced through the classic Fenton reaction between FeSO_4_ and H_2_O_2_ and examined by ESR. Because •OH is a powerful oxidizing agent, it has a short half-life, making it hard to detect. In this study, •OH was captured with the spin trap agent DMPO, forming DMPO/•OOH (31).

ESR spectra showed that the characteristic peak intensities of DMPO/•OH and DMPO/•OOH were markedly decreased with increasing concentrations of PB (**Figure 1I**). A linear correlation was observed between PB concentrations and •OH quenching, with 25 and 50 μg/mL PB concentrations having •OH quenching rates of 32.5% and 56.6%, respectively (**Figure 1J**). These findings showed that PB scavengers quenched •OH in a concentration-dependent manner regardless of Fe composition, showing the good •OH scavenging property. The ROS scavenging properties of PB scavenger may be ascribed to its variable valence state.

#### 3.2.3 POD-Like Activity of PB Scavengers

POD is another type of antioxidant enzyme that can detoxify H_2_O_2_ to H_2_O. H_2_O_2_ can oxidize the colorless compound TMB to the blue-colored oxidized TMB (TMBox) with a maximum absorbance at ~652 nm (32). The POD-like activity of PB scavengers was therefore assessed by measuring the absorbance of TMB. Because the blue color of PB was similar to that of TMBox (33), UV-vis absorbance was performed to ensure that the maximum absorption of oxidized TMB at 650 nm would not be masked by the 20-fold higher concentration of PB (**Figure S6A**). PB showed a concentration-dependent increase in catalytic activity (**Figure S6B**), with higher catalytic activity at slightly acidic pH (**Figure S6C**). To sum up, PB scavengers demonstrated a remarkable ability to scavenge multiple ROS (**Figure 1K**).

### 3.3 PB Scavengers Alleviated Hepatic Ischemia/Reperfusion Injury in Mice

**Figure 2:**
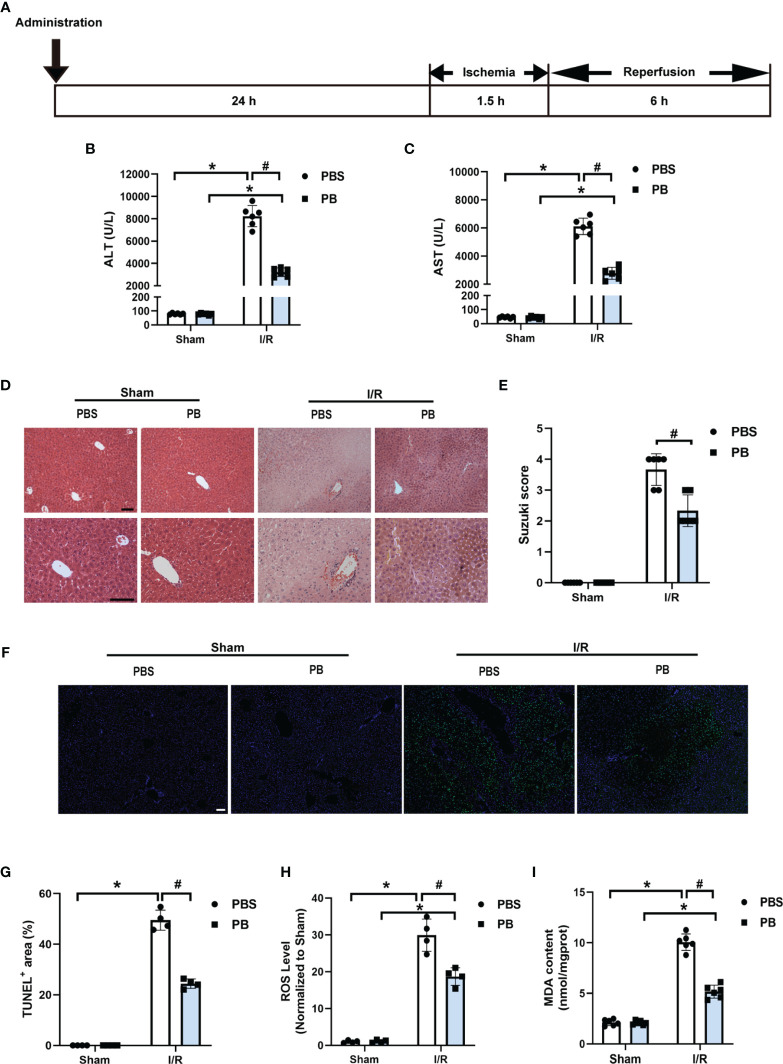
PB scavengers significantly alleviated hepatic ischemia/reperfusion injury in mice. **(A)** Generation of an *in vivo* hepatic I/R injury model in mice. **(B, C)** Serum concentrations of **(B)** ALT and **(C)** AST in mice after 90 min of ischemia and 6 h of reperfusion (n = 6). **(D)** H&E staining of liver tissue harvested from mice administered different treatments. **(E)** Histological severity of hepatic IRI graded using Suzuki’s score (n = 6). **(F)** TUNEL staining of liver sections. **(G)** Quantification of hepatic apoptotic areas in TUNEL-stained liver tissue (n = 4). **(H)** ROS levels of fresh liver tissue from various groups of mice (n = 4). **(I)** MDA levels of liver sections from various groups of mice (n = 6). *P < 0.05 versus the sham group; ^#^P < 0.05 versus the PBS + I/R group. All scale bars = 100 μm.

The therapeutic efficacy of PB scavengers *in vivo* was assessed using a murine HIRI model, in which mice were exposed to ischemia for 90 minutes followed by reperfusion for 6 hours (**Figure 2A**). To determine the optimal timing of 1 mg/kg PB scavenger administrations, PB scavengers were administered at different time points, including 24 hours or 1 hour before the ischemia procedure and at the beginning of the reperfusion. Only prophylactic administration of PB scavengers 24 hours before I/R showed a significant protective effect against tissue damage caused by HIRI, as indicated by reduced serum ALT and AST levels (**Figures 2B, C**, **S7**). Thus, subsequent experiments were performed by prophylactically administrating PB scavengers 24 hours before the procedure.

Histopathological analysis of liver tissue by H&E staining was performed to evaluate damage to liver tissue. The control group of mice, treated with PBS prior to I/R, showed severe congestion, vacuolization and hepatic necrosis, whereas the mice treated with PB before I/R showed only mild or moderate congestion, vacuolization and hepatocyte necrosis (**Figures 2D, E**). Evaluation of hepatocyte apoptosis by TUNEL staining showed that TUNEL positive areas were mainly distributed around the large vessels, with much stronger TUNEL staining in the PBS group than in the PB group after I/R injury, suggesting that PB scavengers could effectively prevent tissue apoptosis (**Figure 2F**). In addition, quantitative analysis confirmed that the area of apoptotic hepatic tissue was larger in the PBS group than in the PB group (**Figure 2G**). Measurement of ROS levels in fresh liver tissue showed that PB scavengers had excellent ROS scavenging capacity *in vivo* (**Figure 2H**). Because lipid peroxidation is an indicator of excessive ROS generation resulting from I/R injury, the levels of MDA, a secondary product of lipid peroxidation, were measured in liver tissue. The level of MDA was markedly higher in the PBS group than in the sham group and the PB group (**Figure 2I**), indicating that PB scavengers could significantly alleviate HIRI injury through inhibition of lipid peroxidation.

### 3.4 PB Scavengers Promoted Macrophage Polarization to M2, Reduced Neutrophil Infiltration and Protected the Liver Against Inflammatory Damage After I/R

**Figure 3:**
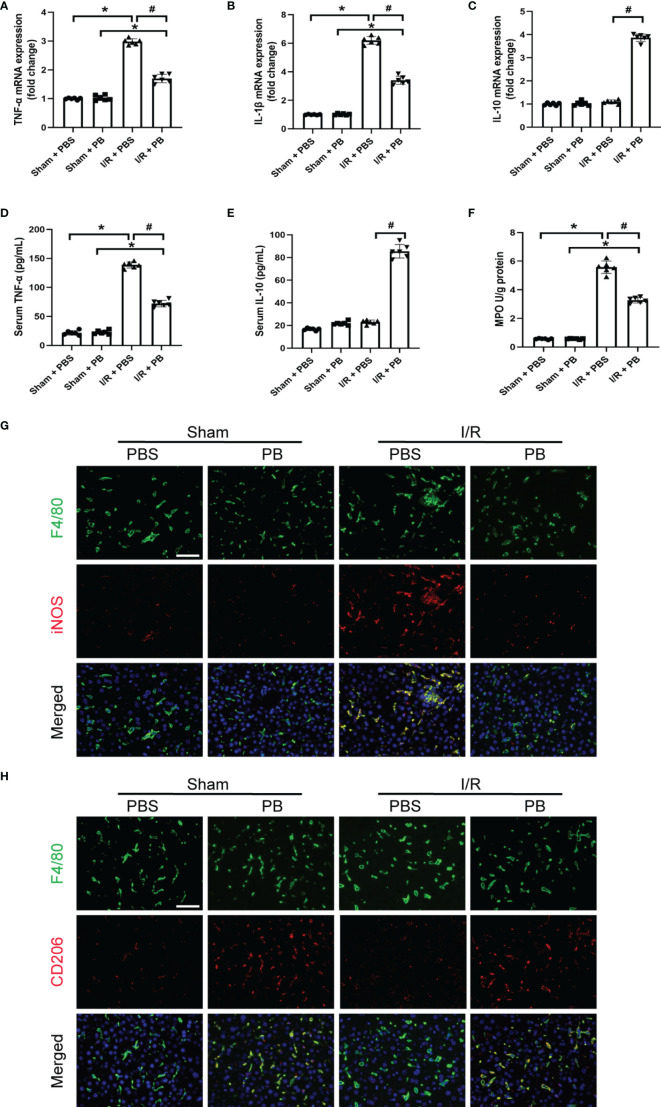
PB scavengers promoted Kupffer cells polarization to M2, reduced neutrophil infiltration and protected liver against inflammatory damage after I/R. **(A–C)** Expression of mRNAs encoding pro-inflammatory (TNF-α, IL-1β) and anti-inflammatory (IL-10) cytokines in mouse liver (n = 6). **(D, E)** Serum concentration of **(D)** TNF-α and **(E)** IL-10 in mice after various treatments (n = 6). **(F)** MPO activity in liver sections of mice after various treatments (n = 6). **(G, H)** Immunofluorescence staining of liver tissue with F4/80 (green), iNOS/CD206 (red), markers of M1 and M2 macrophages, respectively, and the nucleus (blue) in various groups of mice. *P < 0.05 versus the sham group; ^#^P < 0.05 versus the PBS + I/R group. All scale bars = 100 μm.

In addition to high ROS generation, inflammatory response is another strong hallmark of I/R injury. To evaluate the anti-inflammatory activity of PB scavengers in this mouse model of HIRI, the levels of expression of *TNF-α*, *IL-1β* and *IL-10* mRNAs were measured in the livers of PBS- and PB-treated mice. As expected, the levels of expression of the pro-inflammatory *TNF-α* and *IL-1β* were significantly higher in PBS- than in sham-treated mice, but were only slightly higher in PB- than in sham-treated mice (**Figures 3A, B**). The expression of the anti-inflammatory *IL-10* mRNA was significantly higher in PB-treated than in sham- and PBS-treated mice (**Figure 3C**). The anti-inflammatory activity of PB scavengers was further verified by measuring the serum concentrations of TNF-α and IL-10 in these mice (**Figures 3D, E**). MPO activity is often used as a biomarker of neutrophil recruitment in liver tissue after I/R injury (34). Although MPO was significantly higher in PBS-treated mice after I/R injury than in sham-treated mice, MPO activity after I/R injury was markedly decreased by treatment with PB scavengers (**Figure 3F**). Additionally, evaluation of macrophage activation and polarization in the livers of I/R groups by immunofluorescence staining with F4/80, a macrophage/Kupffer cell marker, CD206, an M2 polarization marker and iNOS, an M1 polarization marker, showed that, after the I/R procedure, most of the Kupffer cells in PBS-treated group were activated and had polarized to M1 type, as indicated by the increased number of F4/80^+^iNOS^+^ cells. In contrast, PB scavenger treatment increased the number of F4/80^+^CD206^+^ cells and reduced the number of F4/80^+^iNOS^+^ cells compared with the PBS-treated group (**Figures 3G, H**). These results suggested that PB scavengers might reduce neutrophil recruitment and promote M2 polarization of macrophages, thereby protecting liver tissue against inflammatory damage during I/R.

### 3.5 PB Scavengers Showed Promising Cytoprotective Effect on Oxidative Stress Injury in Primary Hepatocytes

**Figure 4:**
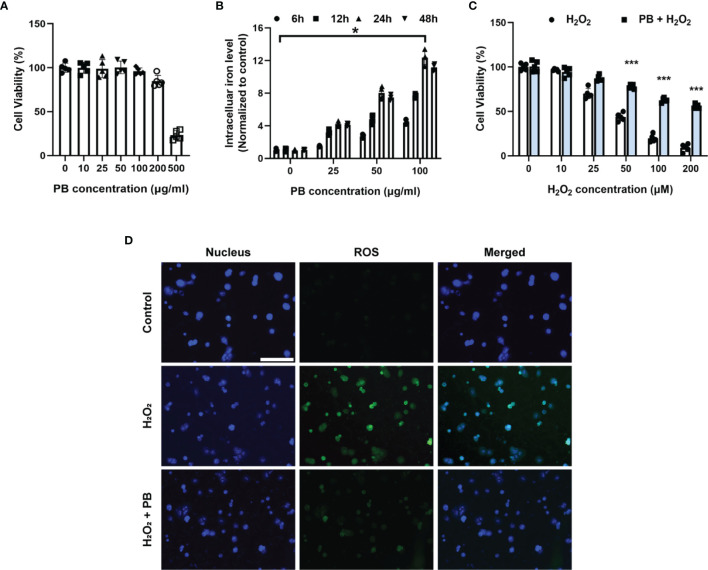
Cytoprotective effect of PB scavengers against oxidative stress injury in primary hepatocytes. **(A)** Viability of primary hepatocytes after treating with different concentrations of PB scavengers for 24 hours (n = 5). **(B)** Intracellular iron levels in primary hepatocytes treated with various concentrations of PB scavengers (n = 4). **(C)** Effect of various concentrations of H_2_O_2_ on the viability of primary hepatocytes in the absence or presence of PB scavengers (50 μg/mL) (n = 5). **(D)** Fluorescence microscopy imaging of intracellular oxidative stress in primary hepatocytes after treatment with H_2_O_2_ (100 μM) for 16 h with or without PB scavengers (50 μg/mL). *P < 0.05 versus control group. ***P < 0.001 versus non-PB-protected groups. All scale bars = 100 μm.

The cytoprotective effects of PB scavengers were also evaluated in primary hepatocytes. At concentrations <100 μg/mL, PB scavengers showed no significant cytotoxicity (**Figure 4A**). Because PB scavenger is an iron-based compound, intracellular iron levels were measured as an indicator of PB uptake by hepatocytes. Intracellular iron concentration increased as PB concentration increased, with intracellular iron peaking at 24 hours of incubation (**Figure 4B**). Primary hepatocytes with or without 50 μg/mL PB scavengers were subsequently treated with various concentrations of H_2_O_2_ to stimulate ROS generation. As expected, increasing H_2_O_2_ significantly reduced cell viability in the absence of PB scavengers, whereas treatment with PB scavengers restored hepatocyte viability (**Figure 4C**). To determine whether the cytoprotective effect of PB scavenger was due to its ROS scavenging ability, intracellular ROS generation in primary hepatocytes exposed to 100 μM of H_2_O_2_ with or without 50 μg/mL PB scavengers was evaluated using DCFH-DA, an ROS-sensitive fluorescent probe. The intracellular fluorescence signal, which was high in the H_2_O_2_ treated hepatocytes, was markedly reduced by PB scavengers, indicating that the latter were efficient scavengers of ROS (**Figure 4D**).

### 3.6 PB Scavengers Alleviated LPS-Induced Inflammation *In Vitro*

**Figure 5:**
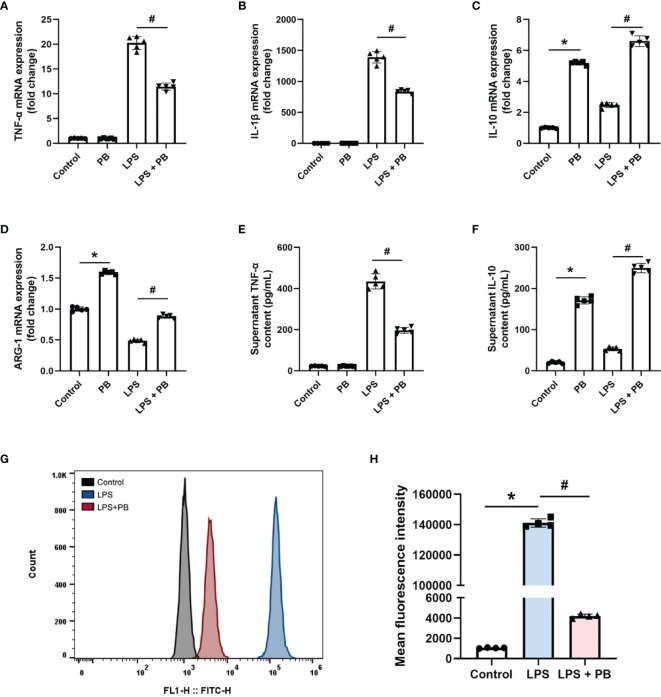
PB scavengers alleviated LPS-induced inflammation in RAW 264.7 cells. **(A–D)** Effect of PB scavengers on the expression of mRNAs encoding the proinflammatory (TNF-α, IL-1β) and anti-inflammatory (IL-10, ARG-1) cytokines in LPS activated RAW 264.7 cells (n = 5). **(E, F)** Secretion of TNF-α and IL-10 by LPS activated RAW 264.7 cells treated with or without PB scavengers (n = 5). **(G)** Flow cytometry analysis of intracellular oxidative stress in the RAW 264.7 cells after LPS activation in the absence or presence of PB scavengers (50 μg/mL). **(H)** Quantitative representation of the flow cytometry results in G (n = 4). *P < 0.05 versus control group; ^#^P < 0.05 versus the LPS-activated group.

The *in vitro* anti-inflammatory properties of PB scavengers were tested using LPS-activated RAW 264.7 macrophage cells. Treatment of LPS-stimulated RAW 264.7 cells with PB scavengers increased the levels of expression of the anti-inflammatory genes *IL-10* and *ARG-1*, but did not affect the expression of the pro-inflammatory genes *TNF-α* and *IL-1β*, compared with control cells, indicating that PB scavengers have anti-inflammatory effects on macrophages (**Figures 5A–D**). In addition, cells treated with LPS and PB scavengers showed significantly reduced levels of expression of *TNF-α* and *IL-1β* and increased levels of expression of *IL-10* and *ARG-1* compared with cells treated with LPS alone (**Figures 5A–D**). Evaluation of the secretion of TNF-α and IL-10 by ELISA showed that TNF-α concentrations were lower and IL-10 concentrations were higher in the supernatants of cells treated with PB scavengers plus LPS than in cells treated with LPS alone (**Figures 5E, F**). Because LPS stimulation of macrophages increased the generation of ROS, which contributed to inflammation, and the over-expression of pro-inflammatory genes may increase the further production of ROS (35), flow cytometry analysis was performed to assess LPS-induced ROS generation in RAW 264.7 cells. As expected, the level of intracellular ROS was higher in LPS-treated than in control cells, whereas PB scavengers reduced the level of LPS-induced ROS (**Figures 5G, H**).

### 3.7 Biosafety of PB Scavengers *In Vivo*

**Figure 6:**
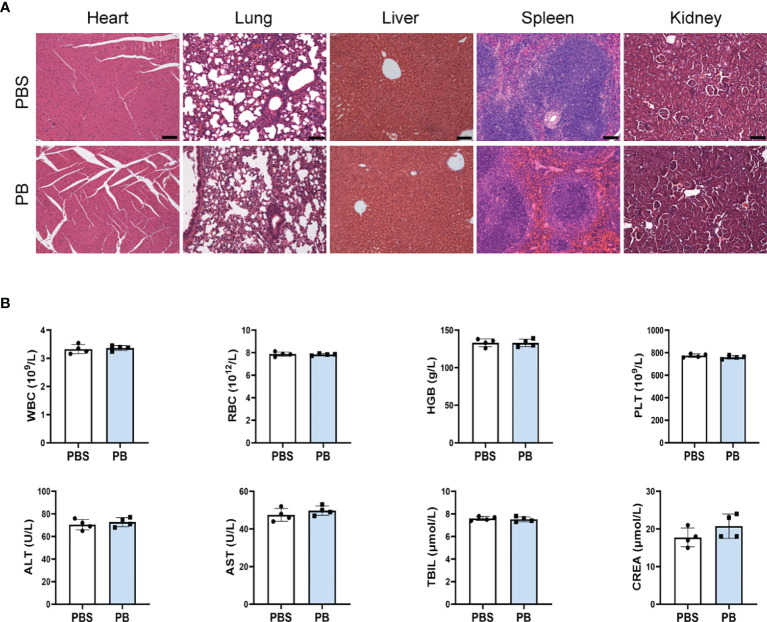
*In vivo* biosafety of PB scavengers **(A)** H&E staining of the major organs of mice administered different treatments. **(B)** Hematological assays of mice 24 h after intravenous injection of PB scavengers (n = 4). All scale bars = 100 μm.

The *in vivo* biosafety of PB scavengers was assessed by H&E staining of various mouse organs. Evaluation of the heart, lungs, liver, spleen and kidneys of PBS or PB-nanozyme treated mice showed no significant tissue abnormalities 24 hours after injection (**Figure 6A**). In addition, PB scavengers had no obvious effects on blood or biochemical indices in these mice (**Figure 6B**).

### 3.8 Discussion

**Scheme 1:**
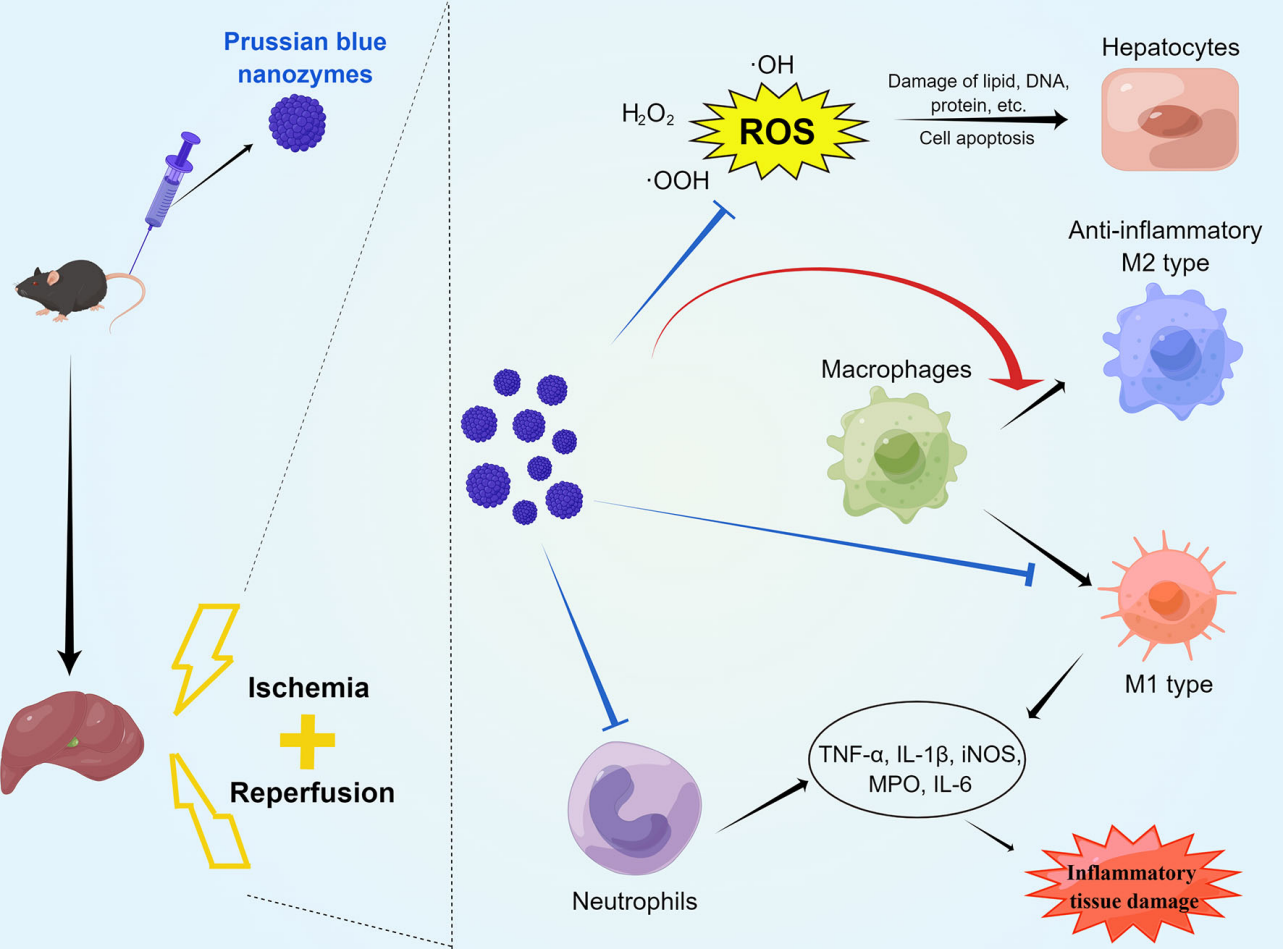
Schematic diagram of the mechanisms by which PB scavengers protect against hepatic ischemia reperfusion injury. Prophylactically administered, PB scavengers can alleviate hepatic ischemia reperfusion injury by scavenging ROS in primary hepatocytes, reducing neutrophil infiltration and promoting macrophage polarization to the anti-inflammatory M2 type. Figure drawn by Figuredraw (www.Figuredraw.com).

The present study found that PB scavengers have promising therapeutic effects on hepatic ischemia reperfusion injury. HIRI injury model mice pretreated with PB scavengers experienced significantly less tissue damage than their positive controls. These nanoparticles with ROS scavenging and anti-inflammatory properties protected the liver of these mice by reducing oxidative stress in hepatocytes, by decreasing neutrophil infiltration, and by promoting macrophage M2 polarization (**Scheme 1**). To our knowledge, the present study is the first to report that systemic prophylactic administration of PB scavengers could protect the liver from acute injury and that systemic use of PB scavengers showed great biocompatibility.

The main strategies presently available to improve outcomes of IRI include reducing oxidative stress in the liver parenchyma and alleviating inflammatory damage (36). Although several drug delivery nanosystems have been designed to target HIRI (37), most are inapplicable clinically because of their poor liver targeting ability or their severe side effects (38, 39). PB scavengers, however, may be effective in the treatment of HIRI. PB itself is an FDA approved drug with high biosafety. Moreover, systemically administered PB scavengers were found to accumulate mostly in the liver and spleen, with little or no obvious tissue damage in the liver, heart, lungs, spleen and kidneys. PB scavenger is nonspecifically taken up by mononuclear phagocyte systems (e.g., liver, spleen), remove these particles from the circulation and resulting in the delivery of a sufficient dose of nano-antioxidants to the liver. In addition to protecting against acute liver injury, PB scavengers have been shown to have good therapeutic effects in ischemic stroke (21), wound healing (24) and inflammatory bowel disease (25), indicating its great potential for treating ROS-associated and inflammatory diseases. Evaluations of its mechanisms of action suggest that PB scavengers reduce intracellular ROS in hepatocytes and macrophages treated with various stimuli, suggesting that these may be critical mechanisms underlying the cytoprotective and anti-inflammatory properties or PB scavengers. ROS are important regulatory signal factors for the M1 polarization of macrophages *via* the downstream NF-κB signal. Suppressing ROS expression can switch polarization from M1 to M2 type (40, 41). PB scavengers were shown to switch macrophage polarization from the pro-inflammatory M1 type to the anti-inflammatory M2 type in the liver, as well as effectively reducing LPS-induced ROS generation in RAW 264.7 cells *in vitro*. These findings indicate that PB scavengers may scavenge intracellular ROS in activated macrophages and Kupffer cells, promoting their M2 polarization. This strategy also offers clues to the application of nanozyme therapy to other liver diseases, such as fatty liver disease and drug-induced liver injury. PB scavengers have also shown other advantageous characteristics, including stability in blood, biocompatibility, biodegradability, and low cytotoxicity. In addition, they are easy to prepare and at low cost; have adjustable morphology and size and have high catalytic activity. These characteristics of PB scavenger can overcome some of the drawbacks of other clinically relevant antioxidants, such as poor solubility, insufficient target specificity, and systemic toxicity. Additional studies are needed to determine the optimum concentration for PB scavenger treatment and methods improving the targeting of PB scavengers to the liver. In addition, PB scavengers may act as drug carriers, combining with other drugs to achieve greater therapeutic effect in ROS-associated and inflammatory diseases.

## 4. Conclusion

This study described the synthesis of a biocompatible ROS scavenger using FDA-approved components. The synthesized PB scavengers effectively protected the liver from IRI by scavenging ROS in hepatocytes and macrophages, reducing apoptosis and alleviating inflammatory damage. Pretreatment with PB scavengers not only improved cell viability under high oxidative stress conditions but promoted macrophages polarization to M2 type and reduced the infiltration of neutrophils. PB scavengers may become a viable and effective treatment option for diseases associated with ROS stress and inflammation.

## Data Availability Statement

The original contributions presented in the study are included in the article/**Supplementary Material**. Further inquiries can be directed to the corresponding authors.

## Ethics Statement

The animal study was reviewed and approved by School of Medicine, Shanghai Jiao Tong University.

## Author Contributions

YH and QXu had full access to all the data in the study and be responsible for the integrity of the data and the accuracy of the data analysis. Study design: KH, QXi and XC. Acquisition of data: YH, QXu, JZ, YY and YZ. Analysis and interpretation of data: YH, QXu and YP. Drafting of the manuscript: YH and QXu. Critical revision of the manuscript for important intellectual content: KH, QXi and XC. Statistical analysis: YH, QXu and JZ. Obtaining funding: KH, QXi and XC. Administrative, technical, or material support: YH, QXu, YY and YP. Supervision: KH, QXi and XC. All authors contributed to the article and approved the submitted version.

## Funding

This study was supported by the Project of the Shanghai Municipal Health Commission (20204Y0012), the Innovative Research Team of High-Level Local Universities in Shanghai (SSMU-ZDCX20180802), the National Natural Science Foundation of China (81972205, 82172074), the Project of Shanghai Key Clinical Specialties (shslczdzk05801), the Seed Fund of Renji Hospital (RJZZ18-010), the Shenkang 3-year action plan (SHDC2020CR2003A, SHDC2020CR5012), Shanghai Rising-Star Program (21QA1407100), and the translational medicine national science and technology infrastructure (Shanghai) open project fund (TMSK-2020-004).

## Conflict of Interest

The authors declare that the research was conducted in the absence of any commercial or financial relationships that could be construed as a potential conflict of interest.

## Publisher’s Note

All claims expressed in this article are solely those of the authors and do not necessarily represent those of their affiliated organizations, or those of the publisher, the editors and the reviewers. Any product that may be evaluated in this article, or claim that may be made by its manufacturer, is not guaranteed or endorsed by the publisher.
