# Supplementary material for: Prussian Blue Scavenger Ameliorates Hepatic Ischemia-Reperfusion Injury by Inhibiting Inflammation and Reducing Oxidative Stress
Source: Front Immunol. 2022 May 25;13:891351. doi: 10.3389/fimmu.2022.891351 (PMC9174572; doi:10.3389/fimmu.2022.891351)
Supplement: Supplementary file 1 [file DataSheet_1.pdf]

Supplementary Material

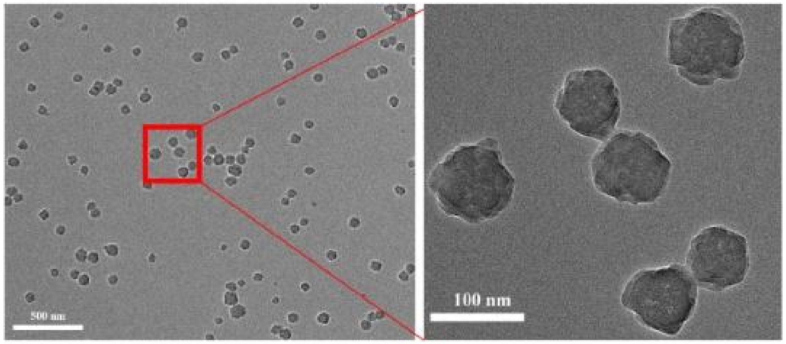

Supplementary Figure 1. TEM image of PB on different scale.

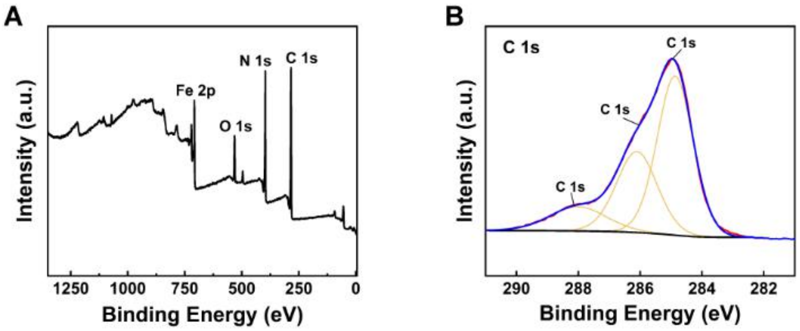

Supplementary Figure 2. XPS spectrum of all elements**(A)** and C 1s**(B)**.

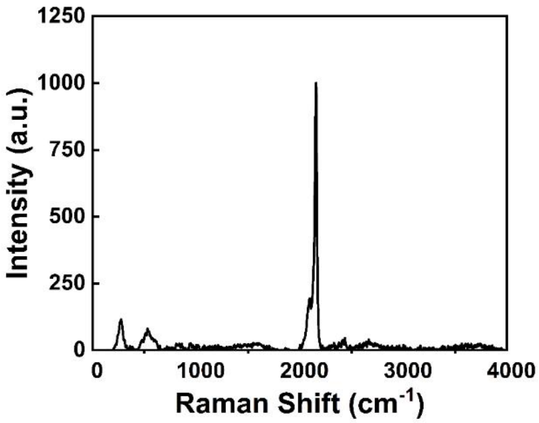

Supplementary Figure 3. Raman spectrum.

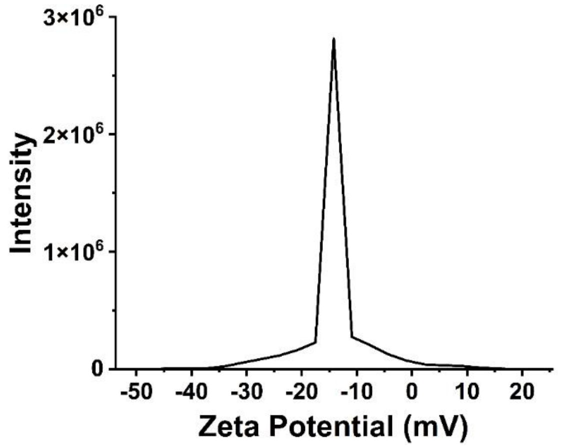

Supplementary Figure 4. Zeta potentials.

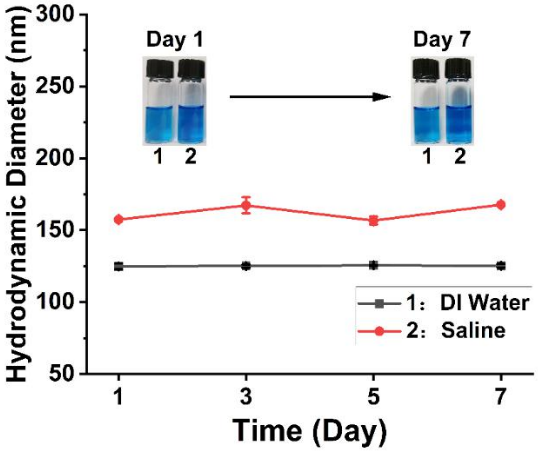

Supplementary Figure 5. Changes in the hydrodynamic size of PB incubated in different media for 7 days.

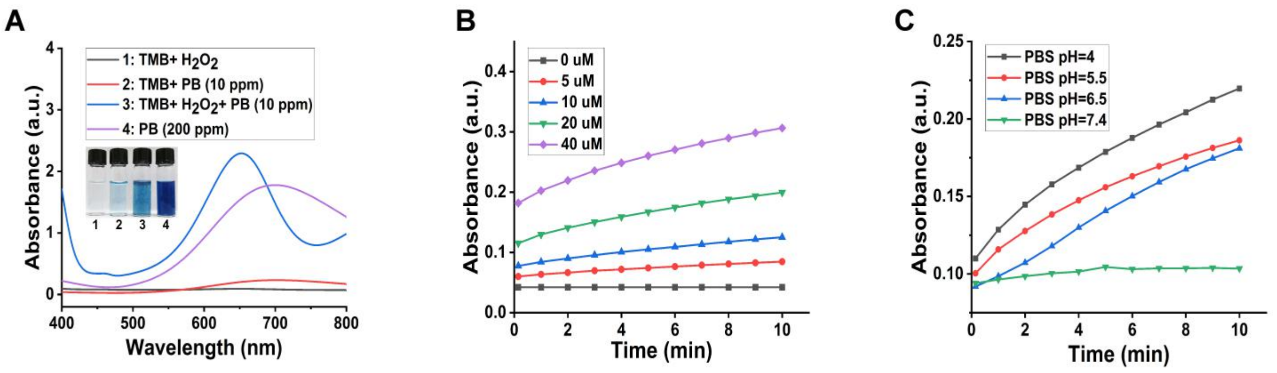

Supplementary Figure 6. POD-like activity of PB. (A) The absorbance curves of TMB/ H_2O_2+ PB of 400-800 nm wavelength (inset: Digital photo of TMB/ H_2O_2+ PB system). The time-dependent absorbance changes of TMB/ H_2O_2+ PB at 650 nm wavelength at various **(B)** PB concentrations (pH=5.5, TMB 100uM, H_2O_2 1mM), **(C)** pH (PB=20uM, TMB 100uM, H_2O_2 1mM).

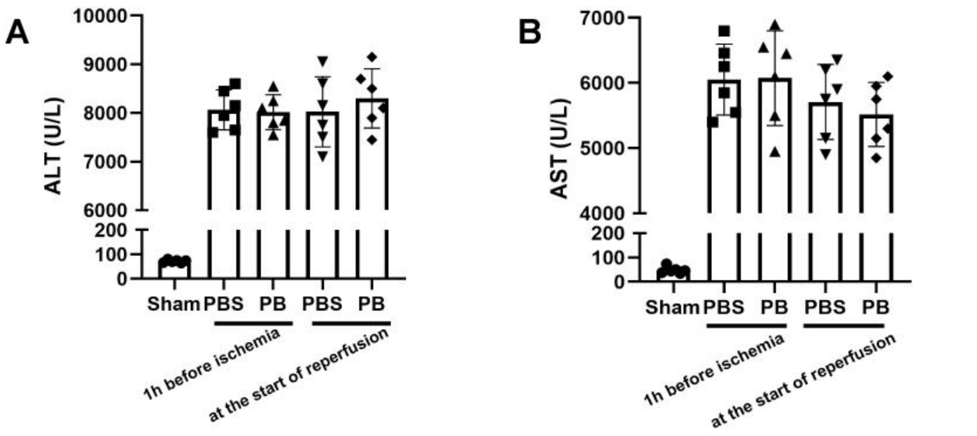

Supplementary Figure 7. Serum levels of ALT (**A**) and AST (**B**) from different treatment groups after 90 min of ischemia and 6 h of reperfusion (n=6).
